# Supplementary material for: A transgenic zebrafish for direct optogenetic activation of FGF/ERK signaling
Source: bioRxiv. 2026 May 13:2026.05.12.724650. Preprint. [Version 1] doi: 10.64898/2026.05.12.724650 (PMC13192925; doi:10.64898/2026.05.12.724650)
Supplement: 1 [file NIHPP2026.05.12.724650V1-supplement-1.pdf]

bioRxiv preprint doi: https://doi.org/10.64898/2026.05.12.724650; this version posted May 13, 2026. The copyright holder for this preprint (which was not certified by peer review) is the author/funder. This article is a US Government work. It is not subject to copyright under 17 USC 105 and is also made available for use under a CC0 license.

560 Supplementary Table 1: List of fixed effects and post-hoc analyses used.

Analysis	Figure	Fixed effects + p-values	Post-hoc analysis
1 – Intensity comparisons	2C	1. Exposure (dark or blue) ($p < 0.0001$)<br>2. Embryo type (Injected, het, or hom) ($p = .00036$)<br>3. Exposure*embryo type ($p = .00036$)	Tukey's Honest Significant Difference (HSD)
2 – Mean-adjusted interquartile range	2D	1. Embryo type ($p < 0.0001$)	Tukey's HSD
3 – Stage comparisons	3B	1. Exposure ($p < 0.0001$)<br>2. Stage ($p < 0.0001$)<br>3. Exposure*Stage ($p < 0.0001$)	Tukey's HSD
4 – Duration comparisons	4B	1. Exposure ($p < 0.0001$)<br>2. Exposure duration ($p < 0.0001$)<br>3. Exposure*exposure duration ($p < 0.0001$)	Student's t-test with a Bonferroni correction

561

562

563 Supplementary Table 2: Corrected P-values from intensity analysis in Figure 2C.

564 (Analysis 1: Linear mixed-effects model with Tukey's HSD to correct for multiple comparisons)

Comparison	P-value
Injected + Dark vs. Injected + Blue	P <0.0001
Heterozygous + Dark vs. Heterozygous + Blue	P <0.0001
Homozygous + Dark vs. Homozygous + Blue	P <0.0001
Injected + Blue vs. Heterozygous + Blue	P <0.0001
Injected + Blue vs. Homozygous + Blue	P = 0.9986
Heterozygous + Blue vs. Homozygous + Blue	P <0.0001

565

566 Supplementary Table 3: Corrected P-values from mean-adjusted interquartile range analysis in

567 Figure 2D.

568 (Analysis 2: Linear mixed-effects model with Tukey's HSD to correct for multiple comparisons)

bioRxiv preprint doi: https://doi.org/10.64898/2026.05.12.724650; this version posted May 13, 2026. The copyright holder for this preprint (which was not certified by peer review) is the author/funder. This article is a US Government work. It is not subject to copyright under 17 USC 105 and is also made available for use under a CC0 license.

**Comparison**	**P-value**
Injected + Blue vs. Heterozygous + Blue	P <0.0001
Injected + Blue vs. Homozygous + Blue	P <0.0001
Heterozygous + Blue vs. Homozygous + Blue	P = 0.9852

Supplementary Table 4A: Dark vs. blue corrected p-values for stage analysis in Figure 3B.

(Analysis 3: Linear mixed-effects model with Tukey's HSD to correct for multiple comparisons)

**Comparison**	**P-value**
Sphere + Dark vs. Sphere + Blue	P = 0.5748
30% epiboly + Dark vs. 30% epiboly + Blue	P <0.0001
Shield + Dark vs. Shield + Blue	P <0.0001
Bud + Dark vs. Bud + Blue	P <0.0001
24 hpf + Dark vs. 24 hpf + Blue	P <0.0001

Supplementary Table 4B: Stage comparison corrected p-values for analysis in Figure 3B.

(Analysis 3: Linear mixed-effects model with Tukey's HSD to correct for multiple comparisons)

**Comparison**	**30% Epiboly**	**Shield**	**Bud**	**24hpf**
Sphere	P <0.0001	P <0.0001	P <0.0001	P <0.0001
30% Epiboly	-	P <0.0001	P = 0.0332	P = 0.0058
Shield	-	-	P <0.0001	P <0.0001
Bud	-	-	-	P = 0.9999

Supplementary Table 5A: Dark vs. blue corrected p-values for kinetics analysis in Figure 4B.

(Analysis 4: Linear mixed-effects model with Bonferroni corrections following Student's t-tests)

**Comparison**	**P-value**
10 min + Dark vs. 3 min + Blue	P = 0.0155
10 min + Dark vs. 7 min + Blue	P <0.0001
15 min + Dark vs. 15 min + Blue	P <0.0001
39 min + Dark vs. 39 min + Blue	P <0.0001
63 min + Dark vs. 63 min + Blue	P <0.0001

bioRxiv preprint doi: https://doi.org/10.64898/2026.05.12.724650; this version posted May 13, 2026. The copyright holder for this preprint (which was not certified by peer review) is the author/funder. This article is a US Government work. It is not subject to copyright under 17 USC 105 and is also made available for use under a CC0 license.

79 min + Dark vs. 79 min + Blue	P = 0.0081

Supplementary Table 5B: Time point comparison corrected p-values for analysis in Figure 4B.

(Analysis 4: Linear mixed-effects model with Bonferroni corrections following Student's t-tests)

**Comparison**	**7 min**	**15 min**	**39 min**	**63 min**	**79 min**
3 min	P <0.0001	P <0.0001	P <0.0001	P = 0.0006	P = 1.0
7 min	-	P = 1.0	P = 1.0	P = 0.0231	P <0.0001
15 min	-	-	P = 1.0	P = 0.1050	P <0.0001
39 min	-	-	-	P = 1.0	P <0.0001
63 min	-	-	-	-	P = 0.0013

bioRxiv preprint doi: https://doi.org/10.64898/2026.05.12.724650; this version posted May 13, 2026. The copyright holder for this preprint (which was not certified by peer review) is the author/funder. This article is a US Government work. It is not subject to copyright under 17 USC 105 and is also made available for use under a CC0 license.

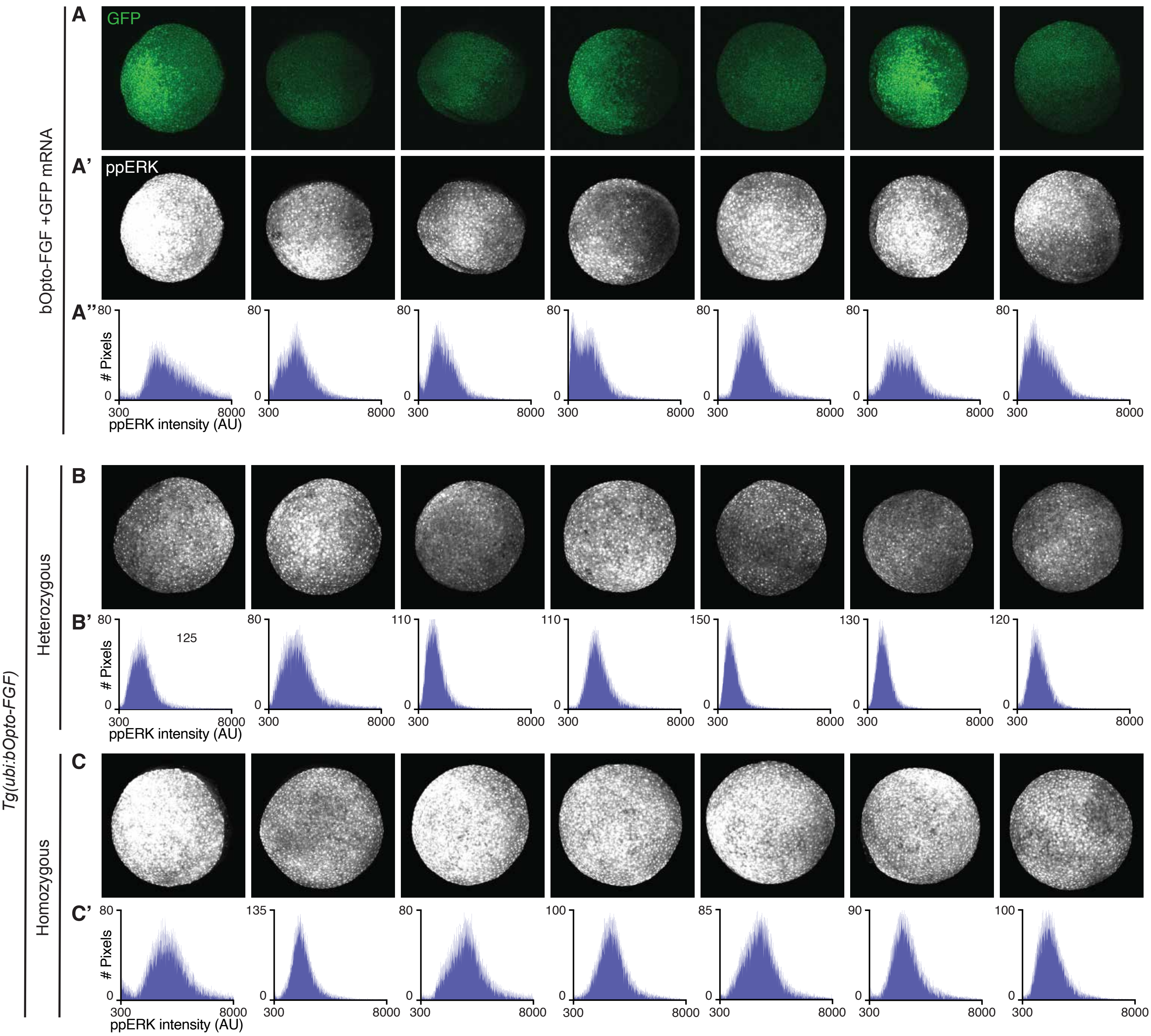

Supplementary Figure 1: Spatial uniformity of optogenetic FGF/ERK signaling activation. *Tg(ubi:bOpto-FGF)* and *bOpto-FGF* + *GFP* mRNA-injected embryos were exposed to 455 nm light (50 W/m^2) for 30 min beginning at shield stage. Embryos were fixed along with unexposed controls. HCR-IF staining for ppERK was used to quantify FGF/ERK signaling. **A-A')** GFP signal (A) and ppERK signal (A') from light exposed mRNA-injected embryos from one trial. **B,C)** ppERK signal from light exposed heterozygous (B) and homozygous (C) embryos from one trial. **A",B',C')** Histograms of ppERK pixel intensities from above images.

bioRxiv preprint doi: https://doi.org/10.64898/2026.05.12.724650; this version posted May 13, 2026. The copyright holder for this preprint (which was not certified by peer review) is the author/funder. This article is a US Government work. It is not subject to copyright under 17 USC 105 and is also made available for use under a CC0 license.

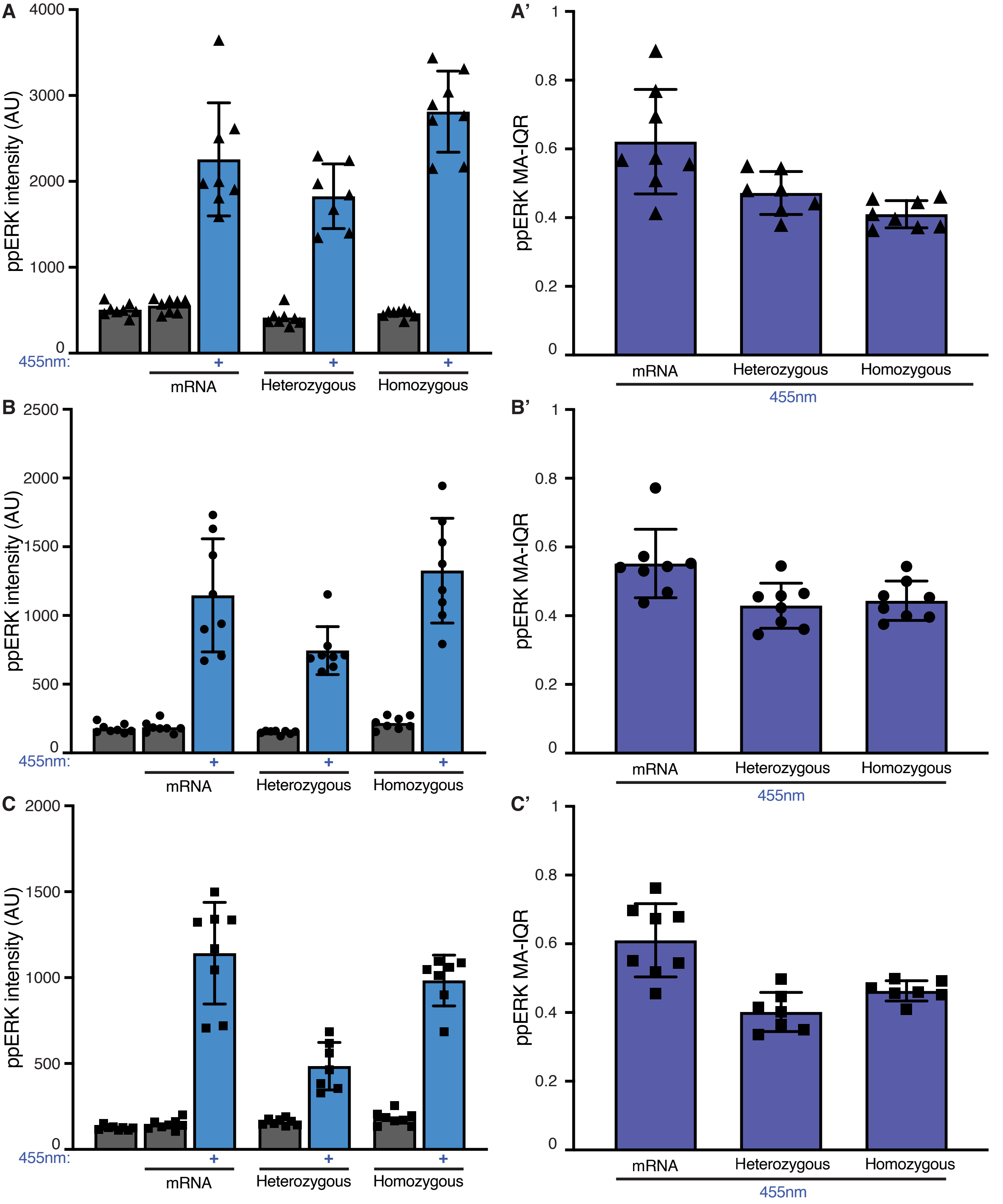

Supplementary Figure 2: Optogenetic FGF/ERK activation independent trials. *Tg(ubi:bOpto-FGF)* and *bOpto-FGF* + *GFP* mRNA-injected embryos were exposed to 455 nm light (50 W/m^2) for 30 min beginning at shield stage. Embryos were fixed along with unexposed controls. mRNA trials also included uninjected, unexposed controls. HCR-IF staining for ppERK was used to quantify FGF/ERK signaling. **A,B,C)** Raw ppERK intensities from three independent trials. Each data point shows the average ppERK intensity from one embryo. The symbol used for each trial is consistent with Fig. 2. Error bars show SD. **A',B',C')** Corresponding ppERK mean-adjusted interquartile ranges (MA-IQR) from the three independent trials shown in A, B, and C. Each data point shows the MA-IQR from one embryo. The symbol used for each trial is consistent with Fig. 2. Error bars show SD.

bioRxiv preprint doi: https://doi.org/10.64898/2026.05.12.724650; this version posted May 13, 2026. The copyright holder for this preprint (which was not certified by peer review) is the author/funder. This article is a US Government work. It is not subject to copyright under 17 USC 105 and is also made available for use under a CC0 license.

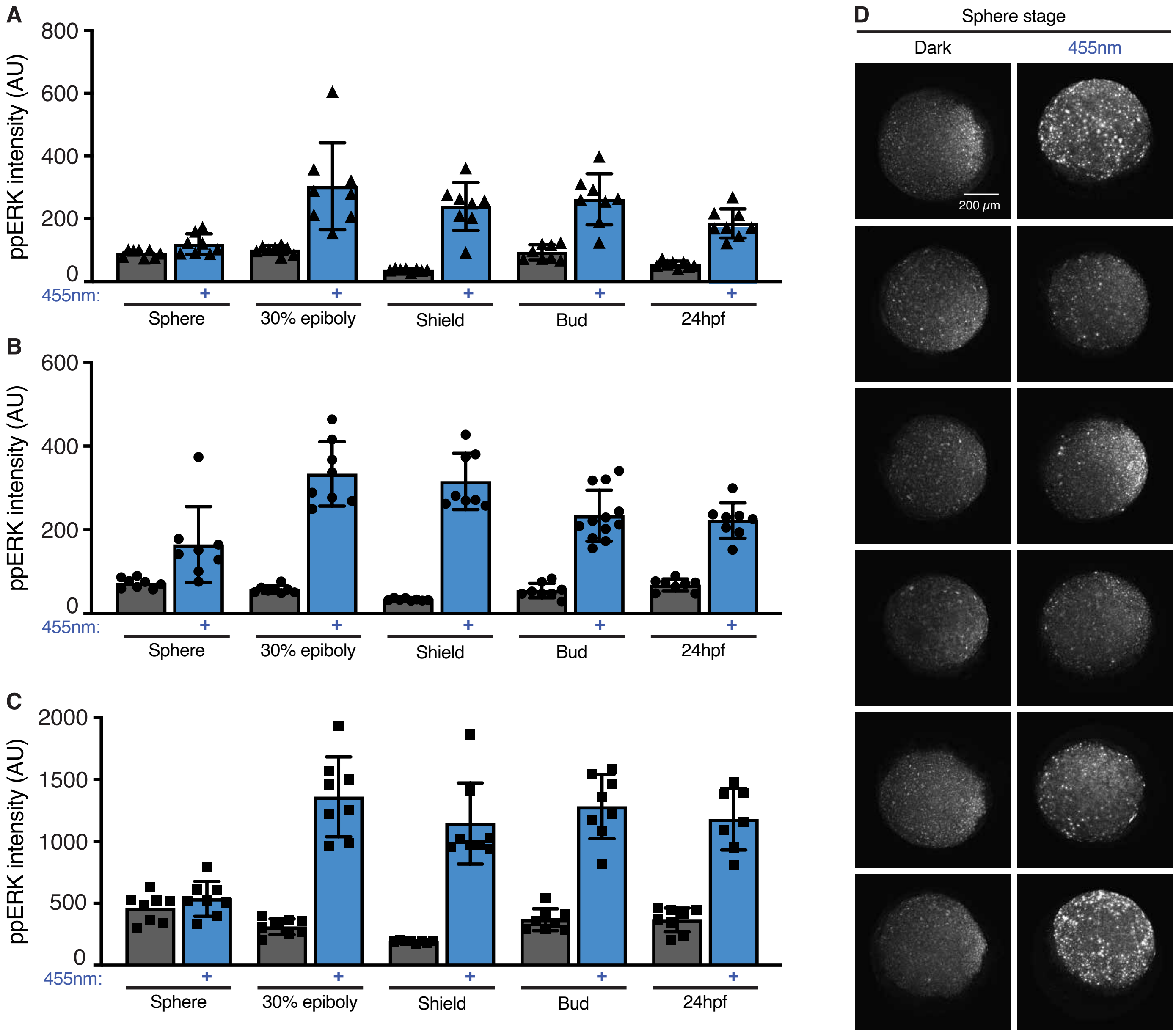

Supplementary Figure 3: Independent timecourse trials & representative sphere stage images. Heterozygous *Tg(ubi:bOpto-FGF)* embryos were exposed to 455 nm light (50 W/m^2) beginning at the indicated stages. Embryos were fixed after 30 min along with stage-matched unexposed controls. HCR-IF for ppERK was used to detect FGF/ERK signaling activity. **A-C)** Raw ppERK intensity data from three independent trials. Each data point shows the average ppERK intensity from one embryo. The symbol used for each trial is consistent with Fig. 3. Error bars show SD. **D)** Representative images of blue light-exposed sphere-stage heterozygous embryos and unexposed controls. Scale bar = 200 μm.

bioRxiv preprint doi: https://doi.org/10.64898/2026.05.12.724650; this version posted May 13, 2026. The copyright holder for this preprint (which was not certified by peer review) is the author/funder. This article is a US Government work. It is not subject to copyright under 17 USC 105 and is also made available for use under a CC0 license.

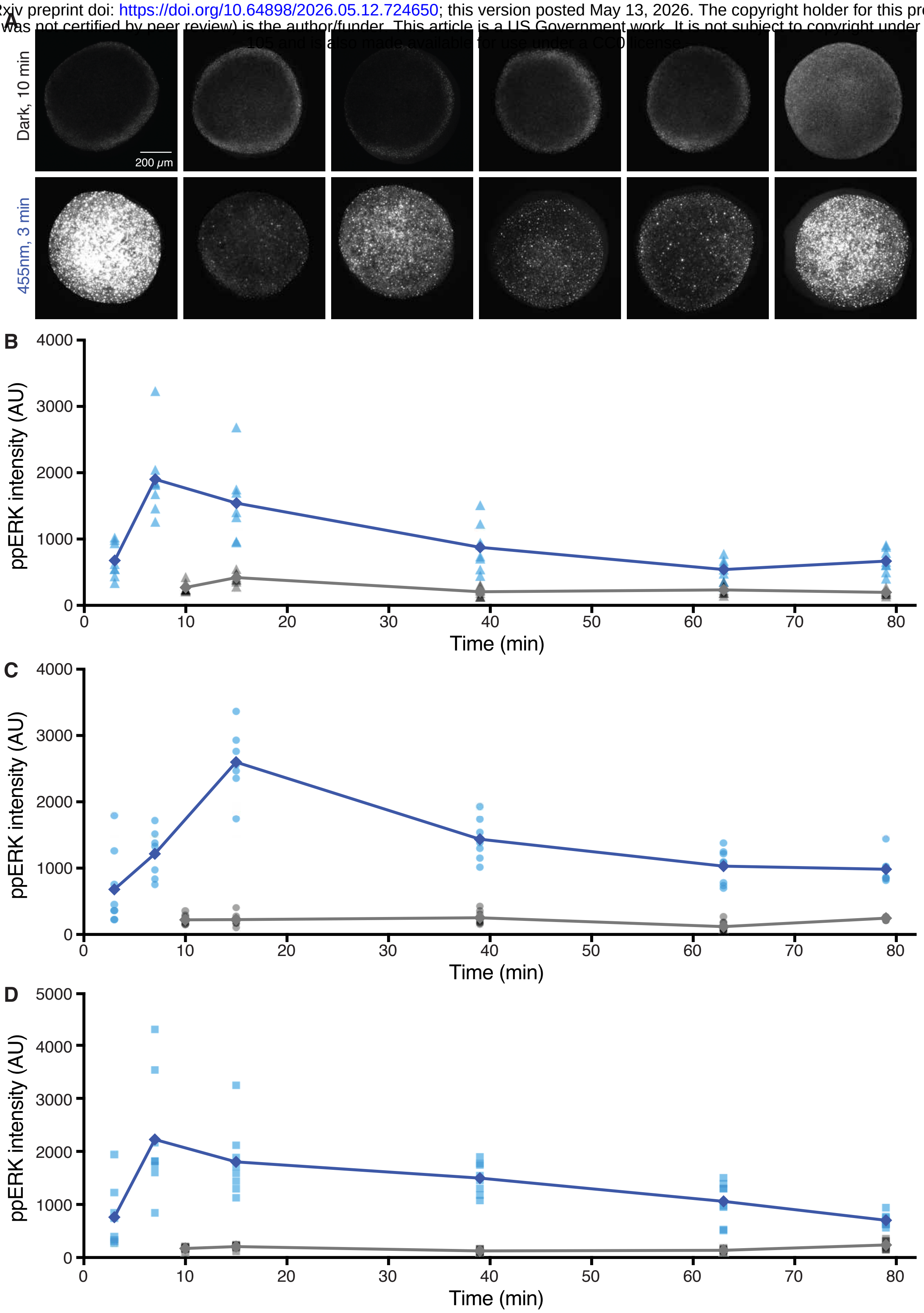

Supplementary Figure 4: Kinetics independent trials & representative 3 min images. Heterozygous *Tg(ubi:bOpto-FGF)* embryos were exposed to 455 nm light (50 W/m^2) beginnging at 30% epiboly. Embryos were fixed along with time-matched unexposed controls at the indicated durations. HCR-IF ppERK staining was used to assess FGF/ERK activity. **A)** Representative images of embryos exposed to blue light and fixed at 3 min (bottom row) and dark controls fixed at 10 min (top row). Scale bar = 200 μm. **B-D)** Raw ppERK intensity data from three independent trials. Each data point (triangle, circle, or square) shows the average ppERK intensity from one embryo. The symbol used for each trial is consistent with Fig. 4. Diamonds represent averaged data.
